# Genotoxicity and Toxicity Assessment of a Formulation Containing Silver Nanoparticles and Kaolin: An In Vivo Integrative Approach

**DOI:** 10.3390/nano13010003

**Published:** 2022-12-20

**Authors:** Adriana Rodriguez-Garraus, María Alonso-Jauregui, Ana-Gloria Gil, Iñigo Navarro-Blasco, Adela López de Cerain, Amaya Azqueta

**Affiliations:** 1 Department of Pharmacology and Toxicology, School of Pharmacy and Nutrition, University of Navarra, Irunlarrea 1, 31008 Pamplona, Spain; 2 Department of Chemistry, School of Sciences, University of Navarra, Irunlarrea 1, 31008 Pamplona, Spain; 3 Navarra Institute for Health Research, IdiSNA, Irunlarrea 3, 31008 Pamplona, Spain

**Keywords:** kaolin, silver nanoparticles, in vivo micronucleus assay, in vivo comet assay, Fpg modified comet assay, genotoxicity

## Abstract

A new material composed of a kaolin base with silver nanoparticles (AgNPs) attached to its surface was developed, as an alternative to antibiotics used as supplements in animal feed. As part of its safety assessment, an in vivo geno-toxicological evaluation of this material was conducted in rats. First, a preliminary dose finding study was carried out to decide the doses to be tested in the main study: 50, 300 and 2000 mg/kg b.w. For the main study, a combined strategy composed of the MN test (TG 474) and the comet assay (TG 489), integrated in a repeated dose 28-day oral toxicity study (TG 407), was performed. A No Observed Adverse Effect Level (NOAEL) of 2000 mg of the silver-kaolin formulation/kg b.w. by oral route, for 28 days, was determined. The silver-kaolin formulation did not induce micronuclei in bone marrow, or DNA strand breaks (SBs) or alkali labile sites (ALS) in liver, spleen, kidney or duodenum at any dose. The modified Fpg comet assay did not reveal oxidized bases in the same tissues at the dose of 2000 mg/kg b.w. Silver was quantified by ICP-MS in all the target organs, confirming the negative results obtained under these conditions.

## 1. Introduction

Antimicrobial resistance is a growing problem that poses very serious world economic and health threats, and the use of long-term and low-dose antimicrobials in animal production as growth promoters is one of the main contributors [1,2,3,4,5,6,7]. The recent banning on the use of antibiotics as growth promoters in animal feed led to the need for new alternatives [8,9,10]. The antimicrobial activity of AgNPs has been widely demonstrated, becoming good alternatives to antimicrobials [11,12,13,14,15,16,17,18]. Moreover, in the last few years the development of materials based on clays with AgNPs attached on their surface has emerged, and it has been demonstrated that they maintain and even potentiate the AgNP antimicrobial activity [19,20,21,22,23]. The immobilization of AgNPs on solid matrixes as kaolin or other clays to obtain silver-clay based materials enhances the AgNP stability, avoids their agglomeration, strengthens their antimicrobial activity by providing a large number of active surface sites and improves their biocompatibility [19,24,25,26]. The antimicrobial efficacy of these materials has also been demonstrated in in vivo studies, suggesting that these materials could be used as a dietary supplement in animal feed [25,27,28,29].

A new material composed by a metallic silver-based clay with bactericidal activity was developed and patented to be used as a feed additive [30]. Perez-Etayo and colleagues demonstrated its in vitro activity against both Gram-positive and Gram-negative bacteria, including resistant and multi-resistant ones to different antibiotic groups, being more effective against Gram-negative bacteria [31]. However, when novel materials are introduced into food sector, it is essential to understand any impact they can cause on animal or human health. Therefore, a safety evaluation is required. The European Food Safety Authority (EFSA) published a guidance on risk assessment for the application of nanoscience and nanotechnologies in the food and feed chain in 2021 [32]. Among the safety evaluation of a compound, the genotoxicity assessment is essential to identify potential mutagens and/or human carcinogens through the detection of primary DNA lesions, gene mutations and chromosomal damage.

In a previous study, an in vitro genotoxicity evaluation of the silver-kaolin formulation was carried out following the strategy suggested by the EFSA guidance, composed by the mouse lymphoma assay, the micronucleus (MN) test and the standard and DNA-formamidopyrimidine glycosylase (Fpg) modified comet assay, obtaining negative results in all of them [32,33]. Negative results in in vitro studies do not usually require going forward with an in vivo evaluation, but regarding this type of complex materials, it is advisable since the current methods have some limitations [17,32,34,35,36,37,38]. 

Thus, the aim of this study was to carry out the in vivo genotoxicity study of silver-kaolin formulation following a strategy based on the suggestions of the EFSA guidance [32]. The evaluation consisted in a preliminary dose finding study, to decide the doses to be tested in the main study. For the main study, a combined strategy was designed, respecting the principles of the 3 Rs (Replacement, Reduction and Refinement). In this way, a combined genotoxicity study composed of the MN test [39] and the comet assay [40], integrated in a repeated dose 28-day oral toxicity study [41] was carried out. 

## 2. Materials and Methods

All the studies were carried out in a laboratory working under Good Laboratory Practices (GLPs) compliance; thus, they were performed under GLPs-like conditions.

### 2.1. Chemicals and Reagents

Physiological saline 0.9%, isoflurane (IsoVet^®^) (Braun, Tuttlingen, Germany), sodium citrate dihydrate (Guinama, Valencia, Spain). Phenylephrine 10%, tropicamide 1% (Alcon Laboratories, Geneva, Switzerland), penicillin-streptomycin (Lonza, Basilea, Switzerland). MMS, FBS, NaCl, trisodium citrate dihydrate, NaOH, KCl, IGEPAL, sucrose, DMSO, citric acid, LMP, standard agarose, Triton X-100, Tris base, HEPES, Na2EDTA, Hartman’s Fixative (Davidson´s Fixative), BSA, DAPI, giemsa, DPX mounting medium, albumin, aspartate amino transferase (AST), alanine amino transferase (ALT), bilirubin (BIL), brilliant Cresil Blue, cholesterol (CHO), triglycerides (TG), creatine phosphokinase (CPK), creatinine (CREA), alkaline phosphatase (ALP), glucose (GLU), protein, urea, calcium (Ca), potassium (K), precinorm 1, precinomr 2, calibrator, ISE low standard, ISE high standard, ISE reference solution, ISE diluent, ISE internal standard, ECO-D, NaOH, NaCl, cellpack (EPK), stromatolyser-4dl (FFD), stromatolyser-4ds (FFS), sulfolyser (SLS), stromatolyser-fb (FBA), control blood (Sysmex). Prothrombin (PTT-a), fibrinogen, Neoplastin Plus, STA calcium chloride 0.025 M, Preciclot Universal I/II, Owren-koller 00360 Limpcub1, Limpcub 2 (Sigma, St. Lluis, MO, USA). Absolute methanol, formaldehyde 4%, ethanol 96% (Panreac, Barcelona, Spain). Fpg (NorGenoTech, Oslo, Norway). Test strips Control-Test^®^ M, test strips Combur 10 Test^®^ M (Roche, Basilea, Switzerland). KBrO_3_, nitric acid 65% p.a., (Merck, Darmstadt, Germany). RPMI-1640 medium containing D-glucose, EMA, HEPES, L-glutamine, sodium bicarbonate and sodium pyruvate (ref. A10491-01), PBS (Gibco-Thermo Fisher Scientific, Waltham, MA, USA).

### 2.2. Test Compound

Silver-kaolin formulation was produced through a method under patent (ENOSAN) and was provided by Laboratorios ENOSAN, Zaragoza, Spain. The material was a formulation composed of a kaolin matrix (68% kaolinite, 12% quartz, 13% illite and 6% potassic feldspar) containing AgNPs (0.83 ± 0.04% (*m*/*m*), average diameter of 27 nm) embedded in its surface. More detailed information about the characterization of the material was provided in a previous study [33]. Silver-kaolin formulation was weighted, and distilled water was added to reach the corresponding concentration. The suspension was then mixed by vortex for 5 min. Just before administration, the suspension was mixed again to ensure a homogeneous dispersion.

### 2.3. Animals

All procedures were approved by the Ethical Committee for Animal Experimentation of the University of Navarra and carried out in accordance with the ethical protocol CEEA 001-20. Eight-week-old Wistar rats of approximately 160 g for females and 243 g for males (weight variation did not exceed ±20%) were purchased from Envigo (Indianapolis, IN, USA). After their arrival, animals were weighted and housed in groups of five in polypropylene cages, at the following environmental conditions (15 air changes/hour, 12 h day/night cycle, 22 ± 2 °C, relative humidity 55 ± 20%). The animals of the dose finding study and the repeated dose 28-day oral toxicity study were allowed to acclimatize for 5 or 12 days, respectively. Animals were provided with ad libitum access to water and controlled access to food.

### 2.4. Dose Finding Study

A group of 5 female rats were used in the study. A total of seven doses of 2000 mg/kg b.w. silver-kaolin formulation were administered orally to each animal, once a day, for seven days, using a gastrointestinal cannula, in a dosing volume of 1 mL/100 g b.w. General symptomatology was deeply observed 30 min, 1, 2, 4 and 8 h after the first administration and daily until the end of the study following the procedure previously described by Irwin [42]. The day previous to sacrifice, all animals were fasted for approximately 15 h. Previous to sacrifice, blood samples were obtained (see below Section 2.5.2. Analytics), and hematology and biochemistry analyses were carried out. Twenty-four hours after the last dose, the animals were sacrificed in a CO_2_ chamber and a pathology study of spleen, heart, liver, kidneys, thymus and ovaries was carried out.

#### Results Evaluation

General symptomatology was compared with the normal values established in the Irwin tests [42]. Both the individual data for each animal and the group mean of the analytical and pathological parameters were evaluated. It was verified whether the data obtained were within the reference control values collected in the historical database of the toxicology laboratory and the data provided by the animal supplier (Envigo, Indianapolis, IN, USA)) for healthy Wistar rats.

### 2.5. Repeated Dose 28-Day Oral Toxicity Study

A repeated dose 28-day oral toxicity study in rodents was carried out following the principles of the OECD TG 407 [41]. Fifty-two rats were used in the study (*n* = 26 female and *n* = 26 male). Animals were randomly divided into (a) 4 groups of 10 animals each (*n* = 5 female and *n* = 5 male) for the principal study; each group received distilled water (negative control) or, 50 mg/kg (low dose), 300 mg/kg (medium dose) or 2000 mg/kg (high dose) b.w. of the formulation, and (b) two groups of 6 animals each (*n* = 3 female and *n* = 3 male) for a potential reversion study; one group received the distilled water (negative control) and the other high dose of silver-kaolin formulation. 

Fresh silver-kaolin formulation suspensions were daily prepared in distilled water and vortexed for proper mixing. The corresponding treatments were administered daily, for 28 days, orally, using a gastrointestinal cannula, in a dosing volume of 1 mL/100 g b.w. Twenty-four hours after the last dose, the animals of the principal study were sacrificed in a CO_2_ chamber. Fourteen days after the last dose, the animals of the reversion study were sacrificed in a CO_2_ chamber. 

#### 2.5.1. Clinical Observations 

General symptomatology was observed weekly, for 28 days or 42 days for the animals of the principal study or reversion study, respectively, following the procedure previously described by Irwin [42]. Besides, all animals were subjected to a fundus revision after pupil dilatation with an indirect ophthalmoscope (Keeler, Inyo County, CA, USA) and a +78-dioptre lens (Volk Optical, Mentor, OH, USA), before the first day of administration. The negative control and high dose groups of the principal study were subjected to another fundus revision at the end of the principal study. Body weight and food consumption were monitored weekly until the end of the study.

#### 2.5.2. Analytics

The day before sacrifice, all animals were fasted and housed in metabolic cages designed for urine harvest, for approximately 15 h. Then, blood samples were obtained previous to sacrifice: (1) from the retro orbicular plexus for hematology and serum biochemistry, and (2) from the tail vein for coagulation. 

Blood samples for hematology were poured into an EDTAK_2_ tube. Analyses were conducted with an automatic hematologic analyzer Sysmex XT-1800i™ (LabX, ON, Canada). Also, blood extensions were obtained from each animal and stained with Brilliant Cresyl Blue to perform the reticulocyte count. Blood samples for serum biochemistry were poured into a separator gel tube and centrifuged (1500× *g*, 10 min, 20 °C). The serum was obtained and analyzed with a biochemical autoanalyzer Cobas c-311(Roche, Basilea, Switzerland). Blood samples for coagulation were poured into a sodium citrate (0.109 M) tube. After centrifugation, serum was obtained and analyzed with a coagulometer STart4 (GMI Inc., Ramsey, MN, USA). The urine was collected and analyzed using Combur 10 Test^®^ M test strips with a Cobas u 411^®^ analyzer (Roche, Basilea, Switzerland).

#### 2.5.3. Pathology

After sacrifice, all animals were subjected to an external palpation and detection of wounds or external abnormalities. Then, a complete necropsy was carried out. Each organ was extracted and “in situ” observed for macroscopic abnormalities; then, they were weighted, processed and carved. The obtained samples were fixed in formaldehyde 4%, with the exception of the testicles, epididymis and the eyes, that were fixed in Davidson fixative and submerged in 70% ethanol for a minimum of 48 h. Also, bone marrow smears were prepared from the right femur bone marrow obtained after centrifugation (5 min, 575 g, room temperature), and they were preserved with a fixative aerosol. Finally, each tissue was descaled, carved, included, cut and stained to obtain the histological preparations for microscopic evaluation. 

#### 2.5.4. Results Evaluation

Results of the treated groups were compared with the negative control values. The mean and standard deviation (SD) of every parameter were obtained for each sex and group of animals. Data from the weight growth, analytics and organ weights of the principal study groups, were first evaluated by the normality Shapiro–Wilk test. The weight growth data were then evaluated by an unpaired T-test in comparison to the negative control group. Regarding analytics and organ weights, if data were normally distributed, they were evaluated using the One-Way ANOVA test. If data were not normally distributed, they were evaluated using the non-parametric Kruskal–Wallis test, and if statistically significant differences were found, the non-parametric Mann–Whitney U test was applied. Statistical analysis was conducted using Stata/IC 12.1 (StataCorp LLC, College Station, TX, USA). Statistical significance was set up at *p* < 0.05. In case of finding statistical differences in the mean of treated groups, the individual data of each animal of the group were analyzed and compared with historical data of the laboratory.

### 2.6. Genotoxicity Studies

The genotoxicity evaluation of the silver-kaolin formulation was carried out with samples of the females composing the principal groups of the 28-day oral toxicity study: MN were counted in erythrocytes of the femur bone marrow samples and comets were evaluated in samples from liver, spleen, kidney and duodenum.

Five females were included as positive control group for MN and comet assays. They were administered with a single intraperitoneal dose of 4 mg/kg b.w. of Mitomycin C (positive MN control), 24 h before sacrifice, and a single oral dose of 200 mg/kg b.w. MMS (positive comet control), 3 h before sacrifice.

For the Fpg-modified comet assay, positive assay controls (frozen TK6 cells treated with 1.25 mM KBrO_3_ for 3 h) were included in each assay. 

#### 2.6.1. Micronucleus Test

The MN test was carried out following the principles of the OECD TG 474 [39]. In the necropsy, bone marrow samples were obtained from one femur of each animal. The femurs were sectioned through trochanters and epicondyles, then centrifuged (827× *g*, 5 min, room temperature), the bone marrow obtained, and the extensions prepared. After 10 min, the extensions were fixed by introducing them in absolute methanol for 10 min. Once fixed, the bone marrow extensions were introduced in Giemsa 10% in PBS, previously filtered with a Whatman grade 1 filter, in low agitation for 10 min. The dye was then removed by gently renewing the content of the bucket with tap water for 2–3 min. Finally, the extensions were introduced in a bucket with type II water for 2 min. Then, the samples were dried up on a Whatman filter upside down for 10 s and face up during 15 min. Finally, samples were analyzed by the eye with an optical microscope, with the ×100 objective, using immersion oil. Polychromatic erythrocytes (PCE), normochromic erythrocytes (NCE) and MN were scored.

##### Results evaluation

To evaluate the hematopoietic toxicity, the rate between the PCE over the total erythrocytes (in a minimum of 500 NCE and PCE counted), was calculated for each animal. Then, the mean PCE rate of the 5 animals composing each group and their corresponding SDs were obtained. To evaluate the genotoxicity, the MN were determined in at least 4000 PCE and MN% was calculated for each animal, by the application of the following formula.

$$\mathrm{MN}\%=\frac{\mathrm{Number}\;\mathrm{of}\;\mathrm{MN}}{\mathrm{total}\;\mathrm{PCE}}\times 100$$

Then, the mean MN% of the 5 animals composing each group and their corresponding SDs were obtained. The mean MN frequencies and EPC rates of the treatment and negative control groups were statistically evaluated by first applying the normality Shapiro–Wilk test. MN% data were normally distributed; therefore, they were evaluated using the parametric One-Way ANOVA test. Hematopoietic data were not normally distributed therefore, they were evaluated using the non-parametric Kruskal–Wallis test. The mean MN% and EPC rate of the positive control group were also subjected to the Shapiro–Wilk normality test. The data were normally distributed, hence they were then evaluated in comparison to the negative control using the parametric two-tailed unpaired t-test. Statistical significance was set at *p* < 0.05. Statistical analysis was conducted using Stata/IC 12.11 (StataCorp LLC, College Station, TX, USA).

#### 2.6.2. Standard and Fpg-Modified Comet Assay 

The standard comet assay was carried out following the principles of the OECD TG 489 [40]. In the necropsy, liver, spleen, kidney and duodenum samples were obtained for the comet assay. Fractions of liver, kidneys, spleen and duodenum were immediately immersed and washed in cold mincing solution (Mg++, Ca++ and phenol red-free Hank’s balanced salt solution supplemented with 20 mM Na2EDTA and adjusted to pH 7.5; just prior use 10% of DMSO was added). Sections of approximately 1 × 1 × 1 mm, 2 × 2 × 2 mm, 2 × 3 × 5 mm and 1.5 cm were cut from spleen, liver, kidney (containing both cortex and medulla) and duodenum, respectively. Duodenum sections were rinsed extensively with cold mincing solution and cut open longitudinally, then lightly scraped with a scalpel (1–2 times) and rinsed again with cold mincing solution. Each sample was sectioned multiple times with a round scalpel blade until a mash of single cells was obtained. Then, each sample was dispersed in 1.5 mL cold mincing solution, obtaining cell suspensions. All samples were immersed in ice until processed.

On the other hand, sections of each organ from the negative control and high dose groups were placed in labelled cryotubes, snap frozen in liquid nitrogen and stored at −80 °C until the Fpg-modified comet assay was performed. Then, frozen tissue samples were pounded with a tissue crusher (pre-cooled at −80 °C) and dispersed in 1.5 mL of cold mincing solution. All samples were immersed in ice until processed. A vial of positive assay control cells was defrosted for each assay. 

Cell suspensions were mixed with 1% LMP agarose (dissolved in PBS), achieving 0.8% LMP agarose. Two drops of 70 µL of cell suspension per slide were placed on pre-coated slides with 1% standard agarose and a 20 × 20 mm coverslip was placed on top of each gel. Two gels per slide were placed on pre-coated slides with 1% standard agarose. For the standard comet assay, one slide was prepared from each of the four organs of each animal. In the case of the Fpg-modified comet assay, two slides were prepared from each of the four organs of each animal and from each assay control (one for the Buffer F incubations and other for the Fpg incubation). After gel solidification, coverslips were removed, and slides were kept immersed for 1–2 h in lysis buffer at 4 °C. Then, the Fpg-modified comet assay slides were washed with Buffer F three times (5 min each). Afterwards, 45 µL of Fpg enzyme (previously titrated by [43]) or Buffer F were added on the corresponding gels, and 22 × 22 mm coverslips were put on top of each gel. Slides were then incubated in a humidified atmosphere, at 37 °C for one hour. Then, the coverslips of the slides of both standard and Fpg-modified comet assays were removed, and slides were immersed in electrophoresis solution for 40 min at 4 °C before performing the electrophoresis (1.2 V/cm, 20 min) also at 4 °C. Finally, slides were neutralized by washing them with PBS followed by distilled water (10 min, each) at 4 °C. 

Gels were stained with 1 mg/mL DAPI solution and comets were analyzed by fluorescent microscope (Nikon Eclipse 50 i, Tokio, Japan), using the image analysis system Comet Assay IV (Perceptive instruments, Bury Saint Edmunds, UK). A total of 150 randomly selected cells were analyzed per slide, 75 cells of each duplicate gel, and the DNA damage indicator used was the tail DNA intensity (% DNA in tail). The median % DNA in tail of the 75 comets analyzed per gel was calculated and then, the mean of both medians of each slide was obtained. In the case of the standard comet assay, the number of highly damaged comets was determined.

##### Results Evaluation

The mean% DNA in tail and its corresponding SDs were obtained for each organ of the five animals composing each group. In the standard comet assay, the % DNA in tail refers to the presence of SBs or ALS. In the Fpg-comet assay, the net Fpg-sensitive sites were calculated by the difference between the % DNA in tail in the slide treated with Fpg and the % DNA in tail in the slide treated with Buffer F. The % DNA in tail of each organ of the silver-kaolin formulation treatment and negative control groups was statistically evaluated by first applying the normality Shapiro–Wilk test. Data were evaluated using the non-parametric Kruskal–Wallis test as they were not normally distributed. The % DNA in tail of each organ of the positive control group was also subjected to the Shapiro–Wilk normality test. Data were normally distributed; hence, they were evaluated in comparison to the negative control group using the parametric two-tailed unpaired t-test. Statistical analysis was conducted using Stata/IC 12.1 (StataCorp LLC, College Station, TX, USA). Statistical significance was set at *p* < 0.05. MIRCA recommendations were followed in these studies [44]. 

### 2.7. Quantitative Analysis by ICP-MS

Female mice organs (liver, spleen, kidney and duodenum) were appropriately cut into representative pieces and dried in stove at 70 °C to constant weight. The dried tissues were accurately weighed to an accuracy of 0.1 mg. Tissue samples were digested with 10 mL sub-boiling nitric acid (distilled from nitric acid 65% p.a.), in an open acid-decomposition system at 80 °C for 12 h. Solutions obtained were then made up to 25 mL with ultrapure deionized water. For each batch of samples, a blank reagent (*n* = 6) was subjected to similar sample digestion procedure. An ICP-MS spectrometer (Agilent 7850, Agilent, Santa Clara, CA, USA) equipped with a Scott-type double-pass quartz spray chamber, concentric glass nebulizer (MicroMist, Agilent, Santa Clara, CA, USA) and a quartz torch (2.5 mm internal diameter injector), was used to determine total silver on a sample solution previously diluted 1:10 with ultrapure water from the acid digestion solution. Operating conditions were optimized daily for maximum sensitivity following the manufacturer’s recommendations. ICP-MS instrumental and analytical parameters are listed in Supplementary Information Table S1.

Measurements were accomplished by direct calibration using working silver aqueous acidified standards, covering a concentration range from 0.01 to 5.0 µg L^−1^, and Indium (In115) at a concentration of 10 μg L^−1^ as internal standard. The peak (*m*/*z*) Ag107 was monitored. Deionized water blank, reagent blank and in-house acid-matched quality control solution (0.074 µg L^−1^) were frequently analyzed with the samples to identify cross-contamination and to provide on-going quality control information (*n* = 9, 0.074 ± 0.001 µg L^−1^). In order to check the accuracy of analytical procedure, an estimation of silver recovery was performed by spiking on all different tissues assayed samples (*n* = 3) at different levels of concentration. The percentage recoveries were satisfactory ranging from 95.8% to 102.6%. Detection limit (LOD) was calculated according to the criteria established by IUPAC (Xb ± 3 s.d.b) as the average of three times the standard deviation of the reagent blank, setting at 0.006 µg L^−1^, equivalent to 0.003, 0.02, 0.03 and 0.02 mg Kg^−1^ when expressed in terms of liver, spleen, kidney and duodenum samples, respectively. 

The mean Ag content of the 5 samples of each tissue and their corresponding SDs were obtained and statistically evaluated by first applying the Shapiro–Wilk normality test. As data were not normally distributed, they were evaluated using the non-parametric Kruskal–Wallis test. Statistical significance was set at *p* < 0.05. Statistical analysis was conducted using Stata/IC 12.1 (StataCorp LLC, College Station, TX, USA).

## 3. Results

### 3.1. Dose Finding Study

All animals survived the 7-day of daily exposure of a 2000 mg/kg b.w. dose of silver-kaolin formulation without showing relevant clinical signs or toxicologically relevant changes in biochemical or hematological parameters. No relevant macroscopic alterations in abdominal and thoracic organs or in the absolute or relative weight of the organs were detected, therefore the histological study was not performed. Results are available in the Supplementary Information: Figure S1 and Tables S2–S5). Consequently, 2000 mg/kg b.w. was identified as the maximum dose to be tested in subsequent toxicity assays of longer duration (Maximum Repeatable Dose, MRD).

### 3.2. Repeated Dose 28-Day Oral Toxicity Study

All animals survived the 28-day exposure and were sacrificed according to the schedule: day 28 for the animals of the main study and day 42 for the animals of the reversion study. The clinical sings of the animals did not show alterations throughout the entire study. Weight rate gain (Supplementary Information, Figure S2) and food consumption were also within the normal limits established by the animal supplier (Envigo, Indianapolis, IN, USA). All the ocular structures evaluated in the fundus examinations as well as the appearance of the media of the negative control and high dose groups, were found to be normal. Therefore, examinations of the other groups were not carried out.

Several analytical parameters showed statistically significant differences in several parameters in comparison with their correspondent negative control values (Supplementary Information, Tables S6–S9). The urinalysis performed did not show relevant alterations. Furthermore, volume, appearance, color, and odor of urine were found to be normal. Macroscopic or microscopic alterations were not observed in any of the studied groups. Thus, the NOAEL was set at 2000 mg/kg b.w. 

### 3.3. Genotoxicity Studies

#### 3.3.1. Micronucleus Test 

**Figure 1:**
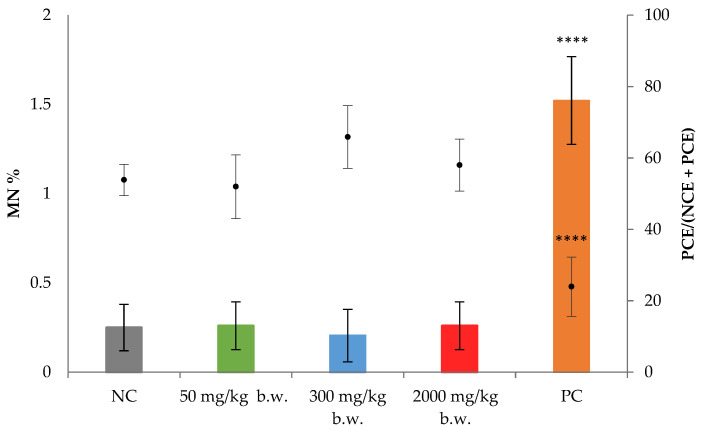
Results of the in vivo erythrocyte MN test after 28-day oral administration of the silver-kaolin formulation. The graphic shows the mean ± SD of the MN% in bars and the PCE ratio over the PCE + NCE (dots) for each group. **** (*p* < 0.001) statistically significant different from negative control. NC: negative control, PC: positive control.

The induction of chromosomal aberrations was assessed by the MN test, in bone marrow samples, obtained from the female rats of the principal groups of the 28-day oral toxicity study, following the principles of the OECD TG 474 (Figure 1) [39]. The ratio of PCE/NCE was determined by counting 500 PCE + NCE. The total MN were determined by counting at least 4000 PCE; then the MN% was determined. 

Mitomycin C at a dose of 4 mg/kg promoted a statistically significant induction of MN in comparison to the negative control, showing mean values of 1.5 ± 0.2% and 0.3 ± 0.1%, respectively. Neither silver-kaolin formulation treatment group showed a significant decrease in the PCE/(NCE + PCE) or a statistically significant increase in MN%.

#### 3.3.2. Standard Comet Assay

**Figure 2:**
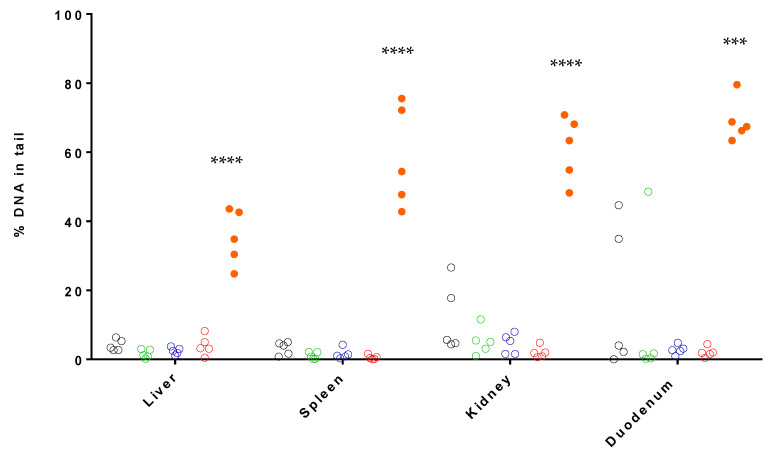
Results of the in vivo standard comet assay in fresh samples of liver, spleen, kidney and duodenum, after 28-day oral administration of the silver-kaolin formulation. The graphic shows the % DNA in tail of each five animals composing negative control (grey), 50 mg/kg b.w. (green), 300 mg/kg b.w. (blue), 2000 mg/kg b.w. (red) and positive control (full orange). *** (*p* < 0.005), **** (*p* < 0.001).

The presence of SBs or ALS was assessed by the standard comet assay in fresh liver, kidney, spleen and duodenum samples, of the female rats of the principal groups of the 28-day oral toxicity study, following the principles of the OECD TG 489 (Figure 2) [40].

MMS, used as a positive control, at a dose of 200 mg/kg b.w. promoted a statistically significant induction of DNA damage than that induced by the vehicle (distilled water), accounting for mean values of 35.23 ± 8.02% in liver, 58.53 ± 14.65% in spleen, 61.08 ± 9.41% in kidney and 69.06 ± 6.18% in duodenum. Negative controls showed values of 4.09 ± 1.66% in liver, 3.21 ± 1.87% in spleen, 11.80 ± 9.98% in kidney and 17.13 ± 20.99% in duodenum. Regarding the results of the silver-kaolin formulation treatments, none of the organs after any of the treatments showed a statistically significant increase in % DNA in tail compared with the negative control. Highly damaged comets were found only in few samples and the values were very low, therefore there were considered as not relevant.

#### 3.3.3. Fpg-Modified Comet Assay 

**Figure 3:**
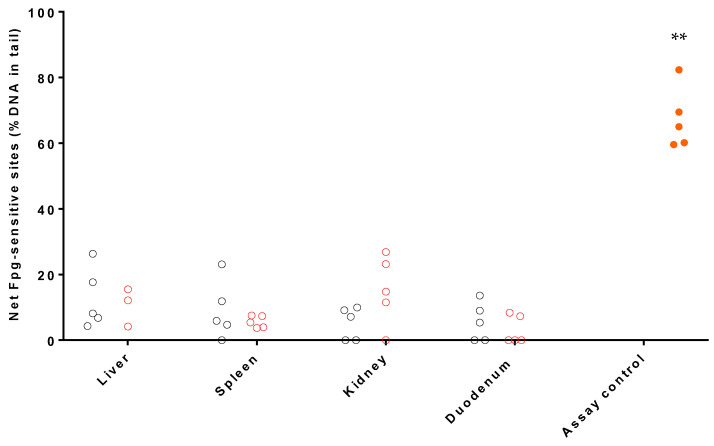
Results of the in vivo Fpg-modified comet assay in frozen samples of liver, spleen, kidney, and duodenum, after 28-day oral administration of the silver-kaolin formulation. The graphic shows the Fpg-sensitive sites in terms of % DNA in tail of each five animals composing negative control (grey), 2000 mg/kg b.w. (red) and assay control (full orange). ** (*p* < 0.01) statistically significant different from negative control.

The induction of Fpg-sensitive sites (i.e., oxidized bases) was assessed by the Fpg-modified comet assay in frozen liver, kidney, spleen and duodenum samples of negative control and high dose groups (Figure 3). In the high dose group, only three % DNA in tail data were obtained to be evaluated in liver, since two of the samples did not contain enough cells to be scored.

TK6 cells treated with 1.25 mM KBrO_3_ for 3 h, used as positive assay control, promoted a net Fpg-sensitive sites according to expectations, accounting for a mean value of 67.30 ± 9.30% DNA in tail. Besides, no significant differences in the induction of Fpg-sensitive sites were found for each tissue in comparison with their respective negative control.

### 3.4. Quantitative Analysis by ICP-MS

**Figure 4:**
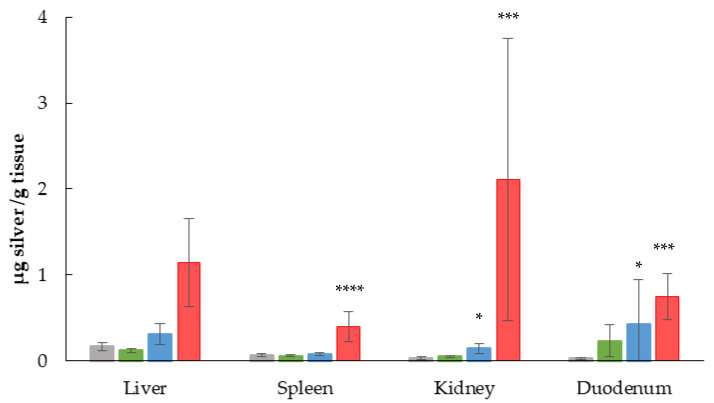
Results of the silver content in frozen samples of liver, spleen, kidney, and duodenum after 28-day oral administration of the silver-kaolin formulation. The graphic shows the mean silver content ± SD of each organ and group (grey: negative control, green: 50 mg/kg b.w., blue: 300 mg/kg b.w., red: 2000 mg/kg b.w.). * (*p* < 0.05), *** (*p* < 0.005), **** (*p* < 0.001).

The amount of silver accumulated in each organ after the administration of 28 oral doses of the silver-kaolin formulation was determined by ICP-MS. Results are shown in Figure 4. Silver was detected and quantified in all the tissues. A statistically significant dose-dependent accumulation was observed in the spleen, kidney and duodenum. Regarding the liver, although the results are not statistically significant, a dose-dependent accumulation trend can be observed.

## 4. Discussion

Kaolin possesses a low toxicity, being the lethal dose 50 (LD50) greater than 5000 mg/kg b.w. in rodents [45,46]. Similar materials have also shown low toxicity in rodent studies [47]. Furthermore, AgNPs of sizes between 10–20 and 8–20 nm presented low toxicity in both acute toxicity and repeated dose toxicity tests, both at doses up to 5000 mg/kg [48,49]. Therefore, the dose to be tested in the preliminary study was set as 2000 mg/kg b.w.

According to the OECD TG 407, at least three test groups and a control group should be used for a repeated dose 28-day oral toxicity study [41]. The high dose was determined in the dose finding study, the low dose was based on an antimicrobial study of a similar formulation in piglets extrapolated to rats [50] and the medium dose was the geometric mean. None of the doses produced mortality or strong adverse effects. Statistical differences were found in some analytical data; however, most of the individual data were within the normal ranges of the laboratory historical values. Altered parameters were not considered relevant from the toxicological point of view when all obtained data about each animal were analyzed together.

It is important to highlight the appropriateness of the design with respect to the 3 Rs principles. The same animals were used to assess the oral toxicity and both genotoxicity endpoints. Moreover, as stated in the OECD TG 489 and OECD TG 474, testing in one single sex is enough for the evaluation [39,40]. The choice of only females in the genotoxicity studies was based on bibliographic evidence of a gender-related AgNP accumulation, being higher in females [51,52,53]. Furthermore, the proper results obtained for both negative and positive controls in all cases are noteworthy. The results obtained demonstrate the robustness of the study and the analytical data obtained from the toxicity study allow their correct interpretation. The presence of silver in the organs evaluated in this study also indicates that the sampling time for the genotoxicity tests was appropriate.

The MN test was carried out with samples of femur bone marrow. The acceptability of the MN test was evaluated applying the OECD TG 474 recommendations [39]. The MN frequencies obtained for the positive and the negative controls were those expected considering the data generated in the laboratory in previous studies. Mitomycin C at a dose of 4 mg/kg b.w. promoted much higher induction of MN than the vehicle of the test product (negative control) and the statistical comparison of these values determined the existence of very significant differences (*p* = 0.009). None of the results obtained for all silver-kaolin formulation doses showed a statistically significant increase in MN in comparison to the negative control, and a dose-related response was not observed.

Liver, kidneys, spleen and duodenum were chosen as target organs for the comet assay as they showed a notable accumulation of AgNPs in previous studies carried out in rodents after repeated administrations [51,52,53,54,55,56,57,58]. The duodenum was also selected as a site-of-contact tissue since the exposure route to silver-kaolin formulation was oral [40,50,59,60,61].

The acceptability criteria of the standard comet assay were those recommended in the OECD TG 489 guideline [40]. The % DNA in tail values obtained for the negative and positive controls were those expected, considering the data generated in the laboratory in previous studies. Regarding the control group, the OECD TG 489 suggests the mean % DNA in tail in rat liver should not exceed a 6% [40]. In this regard, the value obtained for the negative control group was 4.09 ± 1.66%. The results showed little variability, except for two values in kidney and one in duodenum that were much higher than the others (Figure 2). It is worth mentioning that the tissues with high values did not come from the same animals. None of the results obtained for all silver-kaolin formulation doses and tissues showed a statistically significant increase in % DNA in tail, in comparison to their corresponding negative control and a dose-related response was not observed.

The Fpg-modified comet assay does not have an OECD guideline. The levels of Fpg-sensitive sites obtained in the negative control group were in concordance with those previously obtained by the laboratory. Furthermore, the level of Fpg-sensitive sites obtained in the assay controls was as expected (i.e., 67.30 ± 9.30%), which indicated the good performance of the assay. A possible toxicity mechanism of AgNPs is the oxidation, which is the reason why the EFSA guidance recommends including the modified comet assay in the genotoxicity evaluation [32,62,63]. It should be noted that the inclusion of the Fpg-modified comet assay was also performed according to the principles of the 3 Rs. In this case, a positive control itself was not used to reduce the number of animals in the study. For this reason, assay controls were included as positive control for each Fpg-comet assay. An induction of Fpg-sensitive sites was not observed at the highest dose.

According to the EFSA guidance, in order to correctly interpret negative results in the genotoxicity assays, the evidence of the exposure of target organs to the material is essential [32]. Information on the distribution of kaolin is very scarce. Although some studies have evidenced the dissociation of kaolin in the gastrointestinal tract, leading to the distribution and accumulation of aluminum in kidney, liver, heart and brain, in other cases, the accumulation of this metal has been undetectable (reviewed in Maisanaba et al. 2015). Instead, silver is easier to detect; therefore, the accumulation of silver in the organs of the females of the principal study was determined by ICP-MS. Although the amount of silver contained in the bone marrow has not been determined, its accumulation in the liver, spleen and kidney (Figure 4) demonstrates its presence in the bloodstream. Silver circulation in blood carries with it the possibility of causing a genotoxic effect in bone marrow. Furthermore, there is bibliographic evidence proving that AgNPs or the silver contained in them can reach blood circulation and affect bone marrow, after oral administration [51,56]. Thus, it is demonstrated that silver-kaolin formulation did not induce chromosomal aberrations under the tested conditions. A dose-dependent accumulation of silver could be observed in all the organs evaluated. In addition, the kidney stands out among the organs evaluated as the main organ of accumulation. Once the presence of silver in organs was demonstrated after 28 days of administration of the material, it verifies the negative results obtained in the comet assay.

As far as we know, there are no genotoxicity studies of AgNPs-kaolin based materials, but it is known that AgNPs have the capacity to induce chromosomal aberrations and to oxidize DNA bases in vivo [33]. Regarding the studies in which the AgNPs have also been administered orally and at similar schedules, AgNPs of 5 nm, orally administered to mouse once a day, for 35 days, at doses of 10 to 20 mg/kg b.w., resulted positive in comet assay [64]. AgNPs of 20 nm, orally administered to mouse, once a day, for 28 days, at doses of 10 to 250 mg/kg b.w. induced a significant increase of MN in bone marrow [65].

Nonetheless, some safety evaluations of other materials composed by different clays as carriers of AgNPs have been conducted, concluding that clays have very low toxicity and suggesting that the immobilization of AgNPs onto their surfaces reduced silver accumulation in some tissues and improved the safety of AgNPs, while their antimicrobial activity remained [25]. The results obtained in the present study are in agreement with the behavior already observed for other similar materials. The combination of AgNPs attached to the surface of a clay appears to reduce the known toxic effects of silver. Finally, it is also important to highlight the large number of analytical determinations that have been carried out in the toxicity study. No signs of toxic effects were observed in any case. This reinforces the negative results obtained in the genotoxicity tests.

## 5. Conclusions

The NOAEL of silver-kaolin formulation was determined to be a dose of 2000 mg/kg b.w. by oral route, for 28 days. Silver-kaolin formulation did not induce chromosomal aberrations in bone marrow of female rats after 28-day oral administration at any dose. Silver-kaolin formulation did not induce DNA SBs or ALS in liver, spleen, kidney or duodenum of female rats after 28-day oral administration at any dose. Silver-kaolin formulation did not induce oxidized bases in liver, spleen, kidney or duodenum of female rats after 28-day oral administration at doses of 2000 mg/kg b.w. Thus, it can be concluded that the silver-kaolin formulation studied is not mutagenic or genotoxic in vivo, confirming the previous in vitro genotoxicity assessment.

## Data Availability

Not applicable.

## Supplementary Materials

The following supporting information can be downloaded at: https://www.mdpi.com/article/10.3390/nano13010003/s1, Table S1: Instrumental parameters for determination of total silver by ICP-MS Agilent 7850; Figure S1. Results of the dose-finding study weight growth; Table S2. Results of the absolute organ weight for each animal of the dose-finding study (F1-F5); Table S3. Results of the relative organ weight for each animal of the dose-finding study (F1-F5); Table S4. Results of the hematological parameters for each animal of the dose-finding study (F1-F5); Table S5. Results of the biochemical evaluation for each animal of the dose-finding study (F1-F5); Figure S2. Results of the body weight growth from males (a) and females (b) of the 28-day oral toxicity study; Table S6. Results of the hematological evaluation of the repeated-dose 28-day study; Table S7. Results of the absolute and differential count of the repeated-dose 28-day study; Table S8. Results of the biochemical analysis of the repeated-dose 28-day study; Table S9. Results from the coagulation analysis of the repeated-dose 28-day study.
