# Supplementary material for: Genotoxicity and Toxicity Assessment of a Formulation Containing Silver Nanoparticles and Kaolin: An In Vivo Integrative Approach
Source: Nanomaterials (Basel). 2022 Dec 20;13(1):3. doi: 10.3390/nano13010003 (PMC9824684; doi:10.3390/nano13010003)
Supplement: Supplementary file 1 [file nanomaterials-13-00003-s001.zip › nanomaterials-2051980-supplementary.pdf]

Genotoxicity and Toxicity Assessment of a Formulation Containing Silver Nanoparticles and Kaolin: An In Vivo Integrative Approach

Adriana Rodriguez-Garraus [1,*,†], María Alonso-Jauregui [1], Ana-Gloria Gil [1], Iñigo Navarro-Blasco [2], Adela López de Cerain [1,3] and Amaya Azqueta [1,3]

[1] Department of Pharmacology and Toxicology, School of Pharmacy and Nutrition, University of Navarra, Irunlarrea 1, 31008 Pamplona, Spain
[2] Department of Chemistry, School of Sciences, University of Navarra, Irunlarrea 1, 31008 Pamplona, Spain
[3] Navarra Institute for Health Research, IdiSNA, Irunlarrea 3, 31008 Pamplona, Spain
* Correspondence: adriana.rodriguezgarraus@ttl.fi
† Present address: Finnish Institute of Occupational Health, Työterveyslaitos, Topeliuksenkatu 41b, 00250 Helsinki, Finland.

SUPPLEMENTARY INFORMATION

Table S1. Instrumental parameters for determination of total silver by ICP-MS Agilent 7850.

	Parameters	*Setting Values*		*Parameters*	*Setting Values*
Plasma	Plasma mode	General Purpose	*Lens*	Deflect (V)	2.2
	RF power (V)	1550		Cell Entrance (V)	-40
	Nebulizer gas (L min^{-1})	1.06		Cell Exit (V)	-70
	Auxiliary gas (L min^{-1})	0.90	*Cell*	He Gas Flow (mL min^{-1})	4.5
	Plasma gas (L min^{-1})	15.0		Energy discrimination (V)	5.0
	Sampling depth (mm)	10.0		OctP Bias (V)	-18.0
	Spray chamber temperature (ºC)	2.0		OctP RF (V)	190
Lens	Extract 1 (V)	0.0	*QP*	Axis Offset	0.00
	Extract 2 (V)	-170.0		QP Bias (V)	-13.0
	Omega bias (V)	-80	*Torch*	Torch H (mm)	0.5
	Omega lens (V)	9.3		Torch V (mm)	-0.1

Dose-finding study

Weight growth gain

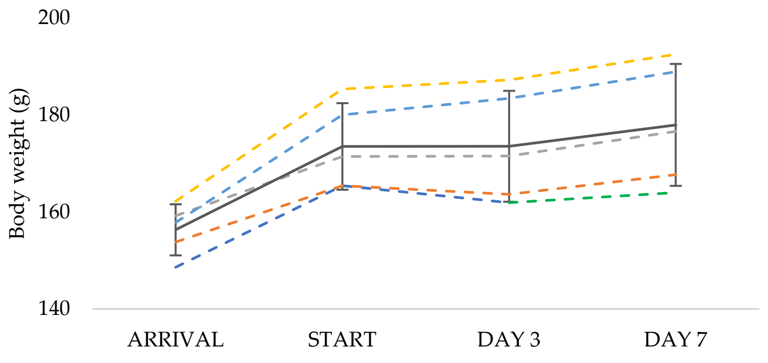

Figure S1. Results of the dose-finding study weight growth. The figure shows the weight growth recording for each study animal, expressed as body weight (g) during the 7 days of the study, represented in dotted lines (F1: green, F2: orange, F3: grey, F4: yellow, F5: blue) and the group mean weight ± the standard deviation (continuous grey line). Acclimatation period between arrival and start lasted 5 days. Start: first day of administration.

Macroscopic alterations

Table S2. Results of the absolute organ weight for each animal of the dose-finding study (F1-F5). The table collects the individual weights (g) of each organ for each animal and the descriptive statistics of the whole group expressed as mean ± standard deviation. The normality ranges of each parameter (bold letter) were calculated from the laboratory histories (n = 57 animals). •: values outside the laboratory records.

Animal ID	Spleen **0.263–0.592**	Heart **0.422–0.945**	Liver **5.068–8.498**	Thymus **0.135–0.568**	Right kidney	Left kidney	Kidneys sum **1.090–1.808**	Right ovary	Left ovary	Ovaries sum **0.060–0.190**
F1	0.350	0.564	4.760•	0.361	0.579	0.584	1.163	0.029	0.042	0.071
F2	0.390	0.498	4.792•	0.401	0.557	0.529	1.086	0.034	0.033	0.067
F3	0.400	0.534	5.710	0.447	0.702	0.673	1.375	0.065	0.069	0.134
F4	0.483	0.592	6.879	0.541	0.761	0.659	1.420	0.064	0.053	0.117
F5	0.411	0.569	5.348	0.562	0.700	0.702	1.402	0.075	0.066	0.141
Mean ± SD	0.407± 0.048	0.551± 0.036	5.498± 0.869	0.462± 0.087	0.660± 0.088	0.629± 0.071	1.289± 0.154	0.053± 0.021	0.053± 0.015	0.106± 0.035

Table S3. Results of the relative organ weight for each animal of the dose-finding study (F1-F5). The table collects the individual weights (g) of each organ for each animal and the descriptive statistics of the whole group expressed as mean ± standard deviation. The normality ranges of each parameter (bold letter) were calculated from the laboratory histories (n = 57 animals). •: values outside the laboratory records.

Animal ID	Spleen **0.138–0.289**	Heart **0.198–0.489**	Liver **2.651–4.128**	Thymus **0.069–0.285**	Right kidney	Left kidney	Kidneys sum **0.546–0.906**	Right ovary	Left ovary	Ovaries sum **0.030–0.096**
F1	0.223	0.360	3.038	0.230	0.369	0.373	0.742	0.019	0.027	0.045
F2	0.247	0.316	3.039	0.254	0.353	0.335	0.689	0.022	0.021	0.042
F3	0.240	0.321	3.431	0.269	0.422	0.404	0.826	0.039	0.041	0.081
F4	0.261	0.320	3.718	0.292•	0.411	0.356	0.768	0.035	0.029	0.063
F5	0.231	0.320	3.011	0.316•	0.394	0.395	0.789	0.042	0.037	0.079
Mean ± SD	0.241± 0.015	0.327± 0.018	3.247± 0.316	0.272± 0.033	0.390± 0.029	0.373± 0.028	0.763± 0.052	0.031± 0.011	0.031± 0.008	0.062± 0.018

Hematological parameters

Table S4. Results of the hematological parameters for each animal of the dose-finding study (F1–F5). The table collects the individual values of each parameter determined for each animal and the descriptive statistics of the whole group expressed as mean ± standard deviation. The normality ranges of each parameter (bold letter) were calculated from the laboratory histories (n = 240 animals).

Animal ID	RBC ($x10^6$cel/µL) **7.53–9.73**	WBC ($x10^3$cel/µL) **3.51–12.05**	Hemoglobin (g/dL) **14.5–17.5**	Htc (%) **41.3–50.1**	MCV (fl) **46.2–59.8**	MCH (pg) **16.9–20.3**	CHMC (g/dL) **32.8–37.2**	Platelets ($x10^3$cel/µL) **582–1203**
F1	8.52	3.73	15.5	43.9	51.5	18.2	35.3	844
F2	8.68	9.42	15.9	43.9	50.6	18.3	36.2	843
F3	8.05	8.64	15.8	45.8	56.9	19.6	34.5	839
F4	8.07	6.24	15.3	43.3	53.7	19.0	35.3	673
F5	8.31	8.28	15.7	44.4	53.4	18.9	35.4	861
Mean ± SD	8.33±0.28	7.26±2.3	15.6±0.2	44.3±0.9	53.2±2.4	18.8±.06	35.3±0.6	812±78

NC: negative control; RV: reversion. (): measurement unit. RBC: red blood cell count, WBC: white blood cell count, Hb: hemoglobin, Htc: hematocrit, MCV: mean corpuscular volume, MCH: mean corpuscular hemoglobin, CHMC: corpuscular hemoglobin mean concentration.

Biochemical parameters

Table S5. Results of the biochemical evaluation for each animal of the dose-finding study (F1-F5). The table collects the group mean and standard deviation of each parameter evaluated. Normality ranges (bold letter) were calculated from the laboratory histories (n = 240 animals). •: values outside the laboratory records.

Animal ID	Albumin (g/dL) **4.0–5.3**	AST (U/L) **39–138**	ALT (U/L) **10–53**	ALP (U/L) **13–138**	Cholesterol (mg/dL) **43–109**	Creatinine (mg/dL) **0.22–0.56**	Total Protein (g/dL) **5.4–7.0**	Urea (mg/dL) **26–59**
F1	4.1	247•	34	110	59	0.38	5.5	34
F2	4.1	74	15	104	54	0.30	5.4	39
F3	4.6	99	22	93	88	0.31	6.0	40
F4	4.2	64	19	94	98	0.33	5.8	34
F5	4.3	193•	32	104	61	0.36	6.0	32
Mean ± SD	4.3±0.2	135±81	24±8	101±7	72±20	0.34±0.03	5.7±0.3	36±3

NC: negative control; RV: reversion; (): measurement unit. AST: aspartate transaminase, ALT: alanine transaminase, ALP: alkaline phosphatase.

Repeated dose 28-day oral toxicity study

Weight growth gain

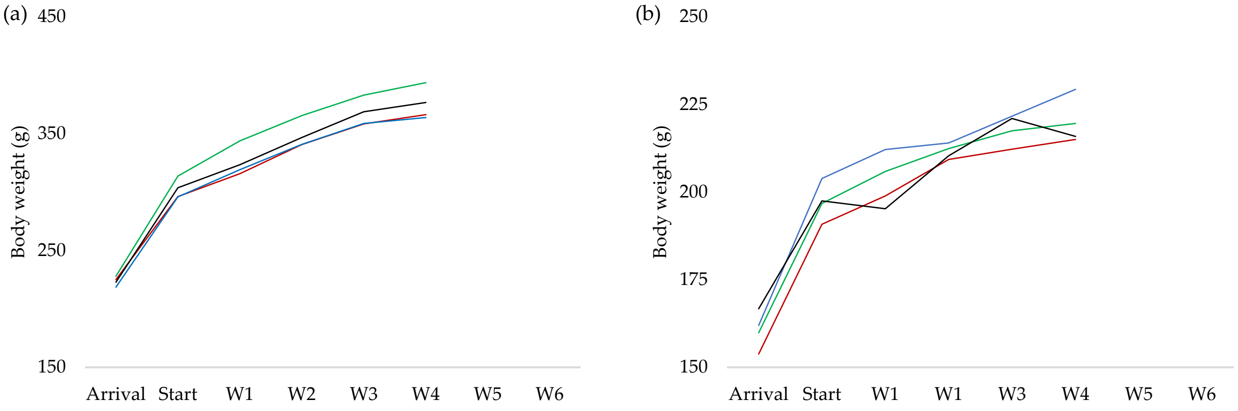

Figure S2. Results of the body weight growth from males (**a**) and females (**b**) of the 28-day oral toxicity study. Each figure shows in lines the mean body weight (g), for each principal study group, throughout the weeks (W) of the study (red: 2000 mg/kg b.w., blue: 300 mg/kg b.w., green 50 mg/kg b.w., black: negative control). Between arrival and start, 12 acclimatization days elapsed. Start: first day of administration.

Hematological parameters

Table S6. Results of the hematological evaluation of the repeated-dose 28-day study. The table collects the group mean ± standard deviation and the statistical significance for each parameter evaluated. According to the levels of significance: (*: p <0.05).

	RBC ($x10^6$ cel/ml)	WBC ($x10^3$ cel/ml)	Hb (g/dl)	Htc (%)	MCV (fl)	MCH (pg)	MCHC (g/dl)	Platelets ($x10^3$ cel/ml)	Ret (%)
Females									
NC	8.24 ± 0.33	5.32 ± 1.24	15.0 ± 0.6	42.8 ± 1.9	51.9 ± 0.7	18.2 ± 0.1	35.0 ± 0.5	803 ± 98	2 ± 1
50 mg/kg	8.05 ± 0.27	3.66 ± 0.92	14.9 ± 0.3	42.1 ± 0.	52.3 ± 1.3	18.5 ± 0.5	35.3 ± 1.0	652 ± 159	1 ± 1
300 mg/kg	8.12 ± 0.20	2.72 ± 1.18*	14.9 ± 0.4	42.3 ± 1.1	52.1 ± 0.5	18.3 ± 0.5	35.2 ± 0.6	733 ± 129	1 ± 0
2000 mg/kg	8.22 ± 0.50	3.71 ± 0.60	15.1 ± 0.7	43.1 ± 2.3	52.5 ± 1.3	18.4 ± 0.8	35.1 ± 1.4	655 ± 135	1 ± 1
Males									
NC	9.17 ± 0.35	5.66 ± 0.94	16.1 ± 0.3	44.6 ± 0.5	48.7 ± 1.4	17.5 ± 0.7	36.1 ± 0.7	712± 59	1 ± 1
50 mg/kg	9.19 ± 0.51	6.24 ± 1.06	16.3 ± 0.7	45.4 ± 2.4	49.5 ± 0.4	17.8 ± 0.2	35.9 ± 0.4	832 ± 72	2 ± 1
300 mg/kg	8.87 ± 0.46	6.68 ± 0.21	16.4 ± 0.6	44.9 ± 1.6	50.6 ± 1.8	18.5 ± 0.6*	36.6 ± 0.2	830 ± 118	1 ± 1
2000 mg/kg	9.44 ± 0.44	6.60 ± 1.09	16.7 ± 0.4	47.2 ± 1.6	50.1 ± 1.4	17.7 ± 0.5	35.4 ± 0.8	683 ± 193	1 ± 1

NC: negative control, (): measurement unit. RBC: red blood cell count, WBC: white blood cell count, Hb: hemoglobin, Htc: hematocrit, MCV: mean corpuscular volume, MCH: mean corpuscular hemoglobin, CHMC: corpuscular hemoglobin mean concentration.

Table S7. Results of the absolute and differential count of the repeated-dose 28-day study. The table collects the group mean ± standard deviation and the statistical significance for each parameter evaluated. According to the levels of significance: (*: p <0.05).

	Eosinophils		Neutrophils		Lymphocytes		Monocytes		Basophils	
	$x10^3$ cell/mL	%	$x10^3$ cell/mL	%	$x10^3$ cell/mL	%	$x10^3$ cell/mL	%	$x10^3$ cell/mL	%
Females										
NC	0.06 ± 0.02	1.4 ± 0.7	0.58 ± 0.24	12.4 ± 4,6	4.00 ± 1,33	82.1 ± 6,3	0.21 ± 0.17	4.1 ± 2.8	0.00 ± 0.00	0.0 ± 0.0
50 mg/kg	0.04 ± 0.01*	1.2 ± 0.4	0.83 ± 0.06	25.4 ± 2,9	2.31 ± 0.48*	69.6 ± 3,4	0.13 ± 0.04	3.9 ± 1.1	0.00 ± 0.00	0.0 ± 0.0
300 mg/kg	0.04 ± 0.01	2.0 ± 1.0	0.49 ± 0.10	22.3 ± 14,2	2.07 ± 1,12*	71.5 ± 16,0	0.11 ± 0.04	4.3 ± 1.4	0.00 ± 0.00	0.0 ± 0.0
2000 mg/kg	0.07 ± 0.02	1.7 ± 0.4	0.64 ± 0.12	16.1 ± 2,8	3.07 ± 0.31	78.0 ± 3,5	0.17 ± 0.02	4.2 ± 0.8	0.00 ± 0.00	0.0 ± 0.0
Males										
NC	0.12 ± 0.11	1.9 ± 1.8	1.19 ± 0.42	19.5 ± 5.7	4.60 ± 1.17	73.6 ± 6.4	0.30 ± 0.12	5.0 ± 1.9	0.00 ± 0.00	0.0 ± 0.0
50 mg/kg	0.08 ± 0.05	1.2 ± 0.6	1.42 ± 0.64	20.9 ± 8.2	4.78 ± 0.99	71.8 ± 9.9	0.40 ± 0.12	6.0 ± 1.5	0.00 ± 0.00	0.0 ± 0.0
300 mg/kg	0.06 ± 0.02	1.0 ± 0.5	0.86 ± 0.12	14.2 ± 3.1	5.01 ± 1.05	79.8 ± 3.3	0.31 ± 0.06	3.9 ± 2.1	0.00 ± 0.00	0.0 ± 0.0
2000 mg/kg	0.09 ± 0.06	1.5 ± 0.9	0.96 ± 0.32	16.6 ± 5.5	4.62 ± 0.99	77.8 ± 7.0	0.23 ± 0.16	4.3 ± 3.4	0.00 ± 0.00	0.0 ± 0.0

NC: negative control; (): measurement unit.

Biochemical parameters

Table S8. Results of the biochemical analysis of the repeated-dose 28-day study. The table collects the group mean ± standard deviation and the statistical significance of each parameter evaluated. According to the levels of significance: (*: p <0.05), (**: p<0.01), (****: p<0.001).

	Females			
	NC	50 mg/kg	300 mg/kg	2000 mg/kg
ALB (g/dL)	4.3 ± 0.2	4.7 ± 0.4	4.2 ± 0.2	4.3 ± 0.2
Urea (mg/dL)	33 ± 4	38 ± 4	34 ± 12	30 ± 2
AST (U/L)	77 ± 10	82 ± 6	89 ± 4*	92 ± 3**
ALT (U/L)	23 ± 2	18 ± 2**	22 ± 2	22 ± 4
ALP (U/L)	108 ± 15	47 ± 6**	106 ± 24	104 ± 17
BIL-T (mg/dL)	0.11 ± 0.03	0.09 ± 0.02	0.10 ± 0.02	0.11 ± 0.03
CHOL (mg/dL)	77 ± 11	74 ± 16	70 ± 5	78 ± 7
GLU (mg/dL)	107 ± 11	111 ± 22	115 ± 22	102 ±16
CREA (mg/dL)	0.34 ± 0.02	0.40 ± 0.08	0.35 ± 0.05	0.34 ± 0.05
TP (g/dL)	6.2 ± 0.3	6.2 ± 0.3	6.0 ± 0.2	6.0 ± 0.3
CPK (U/L)	546 ± 219	525 ± 76	756 ± 100	726 ± 102
Ca (mg/dL)	9.94 ± 0.31	10.20 ± 0.25	10.19 ± 0.18	10.15 ± 0.16
TG (mg/dL)	36 ± 11	54 ± 3	35 ± 10*	37 ± 7
Cl (mg/dL)	93.86 ± 1.24	101.9 ± 1.0****	102.3 ± 0.6****	105.5 ± 5.4****
K (mg/dL)	4.28 ± 0.20	4.50 ± 0.31	4.56 ± 0.31	4.94 ± 0.24
Na (mg/dL)	132 ± 9	145 ± 2*	140 ± 8	145 ± 8*
Glob (g/dL)	2.0 ± 0.2	1.7 ± 0.1	1.8 ± 0.2	1.7 ± 0.1

NC: negative control; (): measurement unit. ALB: Albumin, AST: aspartate transaminase, ALT: alanine transaminase, ALP: alkaline phosphatase, BIL-T total bilirubin, CHOL: total cholesterol, GLU: glucose, CREA: creatinine. TP: total protein, CPK: creatine phosphokinase, Ca: calcium, TG: triglycerides, Cl: chlorine, K: potassium, Na: sodium, Glob: globulin.

Table S8. Cont. Results of the biochemical analysis of the repeated-dose 28-day study. The table collects the group mean ± standard deviation and the statistical significance of each parameter evaluated. According to the levels of significance: (*: p <0.05), (**: p<0.01), (***: p <0.005), (****: p<0.001).

	Males			
	NC	50 mg/kg	300 mg/kg	2000 mg/kg
ALB (g/dL)	4.7 ± 0.3	4.3 ± 0.1	4.8 ± 0.3	4.8 ± 0.4
Urea (mg/dL)	40 ± 7	37 ± 2	37 ± 4	38 ± 4
AST (U/L)	84 ± 17	93 ± 8	72 ± 10*	91 ± 24**
ALT (U/L)	21 ± 4	22 ± 5	18 ± 3	18 ± 5
ALP (U/L)	48 ± 8	92 ± 17****	58 ± 14	47 ± 9
BIL-T (mg/dL)	0.13 ± 0.02	0.11 ± 0.01	0.10 ± 0.02	0.11 ± 0.02
CHOL (mg/dL)	69 ± 4	81 ± 11	75 ± 16	68 ± 18
GLU (mg/dL)	95 ± 12	107 ± 17	103 ± 17	84 ± 10
CREA (mg/dL)	0.43 ± 0.05	0.40 ± 0.08**	0.43 ± 0.05	0.43 ± 0.05
TP (g/dL)	6.4 ± 0.3	6.2 ± 0.2	6.3 ± 0.4	6.5 ± 0.2
CPK (U/L)	504 ± 147	770 ± 153	407 ± 84	633 ± 293
Ca (mg/dL)	9.72 ± 0.43	10.38 ± 0.42	10.39 ± 0.56	10.24 ± 0.12
TG (mg/dL)	36 ± 11	54 ±13*	54 ± 13	54 ± 13
Cl (mg/dL)	95.6 ± 0.8	101.86 ±1.36****	104.72 ± 1.63***	103.94 ± 2.61**
K (mg/dL)	4.02 ± 0.29	3.91 ± 0.13	4.19 ± 0.28	4.28 ± 0.21
Na (mg/dL)	133 ± 3	142 ±1**	135 ± 3	138 ± 4
Glob (g/dL)	1.7 ± 0.2	1.9 ± 0.1	1.6 ± 0.2	1.7 ± 0.2

NC: negative control; (): measurement unit. ALB: Albumin, AST: aspartate transaminase, ALT: alanine transaminase, ALP: alkaline phosphatase, BIL-T total bilirubin, CHOL: total cholesterol, GLU: glucose, CREA: creatinine. TP: total protein, CPK: creatine phosphokinase, Ca: calcium, TG: triglycerides, Cl: chlorine, K: potassium, Na: sodium, Glob: globulin.

Coagulation parameters

Table S9. Results from the coagulation analysis of the repeated-dose 28-day study. The table collects the group mean ± standard deviation and the statistical significance of each parameter evaluated. According to the levels of significance: (*: p <0.05), (**: p<0.01).

	Males			Females		
Group	Fibrinogen (mg/dL)	PT (sg)	aPTT (sg)	Fibrinogen (mg/dL)	PT (sg)	aPTT (sg)
NC	209.6 ± 9.8	17.0 ± 0.8	29.0 ± 4.9	149.2 ± 9.5	16.4 ± 1.1	29.8 ± 5.3
50 mg/kg	215.7 ± 10.6	14.9 ± 0.3**	28.6 ± 1.3	179.2 ± 23.2*	13.9 ± 0.3	31.1 ± 3.5
300 mg/kg	233.6 ± 17.7	16.3 ± 3.0	30.4 ± 13.0	186.0 ± 12.2**	14.4 ± 0.6	24.7 ± 3.4
2000 mg/kg	220.3 ± 19.4	14.8 ± 1.1*	24.6 ± 3.3	139.5 ± 39.4	12.8 ± 1.8*	24.4 ± 9.3

NC: negative control; (): measurement unit. PT prothrombin time, aPTT: activated partial thromboplastin time.
